# The Association of 9 Amino Acids With Cardiovascular Events in Finnish Men in a 12-Year Follow-up Study

**DOI:** 10.1210/clinem/dgab562

**Published:** 2021-08-04

**Authors:** Raimo Jauhiainen, Jagadish Vangipurapu, Annamaria Laakso, Teemu Kuulasmaa, Johanna Kuusisto, Markku Laakso

**Affiliations:** 1 Institute of Clinical Medicine, Internal Medicine, University of Eastern Finland, and Kuopio University Hospital, 70210 Kuopio, Finland; 2 Institute of Clinical Medicine, Internal Medicine, University of Eastern Finland, 70210 Kuopio, Finland; 3 Institute of Biomedicine, Bioinformatics Center, University of Eastern Finland, 70210 Kuopio, Finland

**Keywords:** amino acids, coronary artery disease, ischemic stroke, cardiovascular disease

## Abstract

**Background and Aims:**

To investigate the significance of 9 amino acids as risk factors for incident cardiovascular disease events in 9584 Finnish men.

**Materials and Methods:**

A total of 9584 men (age 57.4 ± 7.0 years, body mass index 27.2 ± 4.2 kg/m^2^) from the Metabolic Syndrome in Men study without cardiovascular disease and type 1 diabetes at baseline were included in this study. A total of 662 coronary artery disease (CAD) events, 394 ischemic stroke events, and 966 cardiovascular disease (CVD; CAD and stroke combined) events were recorded in a 12.3-year follow-up. Amino acids were measured using nuclear magnetic resonance platform.

**Results:**

In Cox regression analysis, phenylalanine and tyrosine were significantly associated with increased risk of CAD and CVD events, and phenylalanine with increased risk of ischemic stroke after the adjustment for confounding factors. Glutamine was significantly associated with decreased risk of stroke and CVD events and nominally with CAD events. Alanine was nominally associated with CAD events.

**Conclusion:**

We identified alanine as a new amino acid associated with increased risk of CAD and glutamine as a new amino acid associated with decreased risk of ischemic stroke. We also confirmed that phenylalanine and tyrosine were associated with CAD, ischemic stroke, and CVD events.

Cardiovascular disease (CVD), including coronary artery disease (CAD) and ischemic stroke, is the leading cause of mortality worldwide (1). Multiple risk factors accelerate the development of CVD, including age, dyslipidemia, hypertension, smoking, hyperglycemia, and inflammatory factors (2-6). However, overall predictive value of the conventional cardiovascular risk factors is limited, and therefore there is an increasing need to identify novel risk factors for senility CVD (5, 6).

Several previous studies on amino acids have especially focused on their associations with obesity (7) and type 2 diabetes (T2D), a major risk factor for CVD (8-10). Previously published studies on the association of amino acids with CAD, ischemic stroke, and CVD (11-19) have been heterogeneous with respect to size of the study (small, large), study design (cross-sectional case-control studies, prospective studies), endpoints (CAD, ischemic stroke, or combination of both).

T2D, CAD, and ischemic stroke share several risk factors, but the role of amino acids remains unclear in the development of cardiovascular events. Previous prospective studies have not systematically compared the significance of amino acids as predictors of CAD, stroke, and CVD in a large prospective population-based study. Therefore, the aim of our study was to investigate the association of serum concentrations of 9 amino acids (alanine, glutamine, glycine, histidine, isoleucine, leucine, phenylalanine, tyrosine, and valine), with the risk CAD, ischemic stroke, and CVD in a large longitudinal population-based Metabolic Syndrome in Men (METSIM) study (20).

## Study Population

The METSIM study includes 10 197 men, aged from 45 to 73 years, and randomly selected from the population register of Kuopio, Eastern Finland. At baseline, each participant had an outpatient visit to the Clinical Research Unit at the University of Kuopio. The design and methods of the METSIM study have been previously described in detail (21).

A total of 9584 men including 1412 men who had T2D were included in the current study. Participants with type 1 diabetes (n = 25) were excluded from current analyses. Altogether, 602 CVD events before the baseline study were recorded, including 404 CAD and 198 ischemic stroke events. These participants were excluded from all statistical analyses. We measured height and weight to the nearest 0.5 cm and 0.1 kg, respectively. Body mass index (BMI) was calculated as weight (kilograms) divided by height (meters) squared. We measured waist circumference at the midpoint between the lateral iliac crest and lowest rib. Smoking status was defined as current smoking (yes vs no). Diagnosis of hypertension was based on the use of antihypertensive medication. All participants underwent a 2-hour oral glucose tolerance test (75 g of glucose), and samples for plasma glucose and insulin were drawn at 0, 30, and 120 minutes. Diabetes was defined as fasting plasma glucose ≥7.0 mmol/L and/or 2-hour plasma glucose ≥11.1 mmol/L, and/or hemoglobin A1c ≥6.5%) and/or medication for diabetes according to the American Diabetes Association 2003 criteria (22).

## Calculations

We validated the best oral glucose tolerance test-derived index of insulin sensitivity in a separate sample of 287 nondiabetic individuals from the region of Kuopio against the euglycemic-hyperinsulinemic clamp, a gold standard measurement of insulin sensitivity (21). Among 6 different indices tested the Matsuda insulin sensitivity index (Matsuda ISI) had the highest correlation 0.776 with the M value from the clamp. We calculated Matsuda ISI for insulin sensitivity based on glucose and insulin measurements at 0, 30, and 120 minutes as previously reported (21).

## Laboratory Measurements

We measured plasma glucose, insulin, total triglycerides, low-density lipoprotein cholesterol (LDL-C), high-density lipoprotein cholesterol (HDL-C), and serum high-sensitivity C-reactive protein (hs-CRP), as previously described (23). We measured serum metabolites and lipoprotein lipids using a high-throughput serum nuclear magnetic resonance (NMR) platform operating at 500 MHz. Fasting serum samples collected at the baseline study were stored at -80°C and thawed overnight in a refrigerator before sample preparation. Aliquots of each sample (300 μL) were mixed with sodium phosphate buffer (300 μL). Details of the NMR experimentation have been described previously (24, 25).

## Diagnosis of CAD and Ischemic Stroke Events

We identified nonfatal and fatal CAD and ischemic stroke events from the hospital discharge registry of Kuopio University Hospital, which is the only hospital and cardiology outpatient clinic treating patients in the Kuopio region. Thereafter, 2 cardiologists (R.J., J.K.) reviewed medical records of all subjects with diagnosis of CAD based on the codes I22-I24 in the International Classification of Diseases according to internationally accepted criteria (26). Causes of CAD death were confirmed from death certificates obtained from the National Causes-of-Death Register of Statistics Finland. We identified 662 nonfatal or fatal CAD events, 394 nonfatal or fatal ischemic stroke events based on the codes I63-I69 in the International Classification of Diseases, and 966 nonfatal or fatal CVD events (CAD and stroke events combined). Incident cases of CVD events were recorded from the baseline study visit until February 28, 2021, with a mean follow-up period of 12.3 years.

## Statistical Analyses

We performed statistical analyses using SPSS version 27 (SPSS, Chicago, IL). We log-transformed all continuous variables for statistical analyses, except for age and LDL-C. We used independent samples *t* test and χ ^2^ test to assess statistical significance between the 2 groups. We applied Cox regression analysis to evaluate baseline concentrations of the nine amino acids as predictors for CAD, ischemic stroke, and CVD events. Results of Cox regression analyses are presented without and with adjustment for confounding factors (age, diabetes status, fasting insulin, smoking, BMI, LDL-C, HDL-C, statin treatment, drug treatment for hypertension, and hs-CRP). Hazard ratio (HR) values are shown as per 1 SD increase in amino acid concentrations. The association with outcomes could be reasonably fit by a linear model. We applied a C-index (27) that is a standard measure of the predictive accuracy of a logistic regression model. To calculate C-index we first included confounding factors listed above in the Cox model (model 1) and then we added in the Cox model 3 amino acids (phenylalanine, tyrosine, alanine) (model 2). Correlations were calculated by Pearson correlations. Bonferroni-corrected limit for statistically significance was *P* < 0.0056 given 9 amino acids included in statistical analyses. *P* < 0.05 was considered as nominally significant.

## Ethics Statement

The study protocol was approved by the Ethics Committee of University of Eastern Finland and Kuopio University Hospital. The study was conducted in accordance with the Helsinki Declaration. All study participants gave written informed consent.

## Results

Clinical and laboratory characteristics of the METSIM study participants at baseline are shown in Table 1. Participants with CAD, stroke, or CVD had more often CVD risk factors, including smoking, type 2 diabetes, hyperglycemia, obesity, medication for hypertension, elevated LDL-C, and elevated hs-CRP compared with participants without CVD. Participants with CAD or CVD had elevated concentrations of alanine, isoleucine, leucine, phenylalanine, and tyrosine, and lower concentrations of glutamine than participants without CVD. Supplementary Table 1 shows the correlations of amino acids with clinical and laboratory parameters (28). Alanine (except for LDL-C), isoleucine, leucine, phenylalanine, tyrosine (except for LDL-C), and valine (except for systolic blood pressure and LDL-C) correlated significantly with BMI, waist circumference, systolic blood pressure, LDL-C, total triglycerides, fasting plasma glucose and 2-hour plasma glucose, fasting plasma insulin, 2-hour plasma insulin, hs-CRP, and inversely with Matsuda ISI insulin sensitivity index.

**Table 1.** Clinical and laboratory characteristics including amino acid measurements of 9548 participants at the baseline study of the METSIM cohort

Variables	No CVD (N = 8578)	CAD (N = 666)	Stroke (N = 394)	CVD (N = 970)
Age, y	57.0 ± 7.0	60.6 ± 6.7^*a*^	61.8 ± 6.7^*a*^	60.8 ± 6.8^*a*^
Type 2 diabetes, % of total	5.9	14.0^*a*^	15.2^*a*^	13.3^*a*^
Current smokers, % of total	17.6	24.9^*a*^	20.1	23.5^*a*^
Medication for hypertension, %	19.3	31.0^*a*^	30.7^*a*^	29.6^*a*^
Body mass index, kg/m^2^	27.2 ± 4.1	28.2 ± 4.5^*a*^	27.9 ± 4.6	28.0 ± 4.5^*a*^
Waist, cm	98.4 ± 11.4	101.7 ± 12.3^*a*^	100.9 ± 12.0^*a*^	101.17 ± 12.1^*a*^
Systolic blood pressure, mmHg	137.6 ± 16.4	143.5 ± 18.0^*a*^	144.0 ± 19.1^*a*^	143.5 ± 18.2^*a*^
Diastolic blood pressure, mmHg	87.4 ± 9.3	88.3 ± 10.5	87.5 ± 10.2	88.25 ± 10.4
HDL cholesterol, mmol/L	1.45 ± 0.39	1.36 ± 0.39^*a*^	1.40 ± 0.40	1.38 ± 0.40^*a*^
LDL cholesterol, mmol/L	3.36 ± 0.88	3.38 ± 0.94	3.25 ± 0.99	3.36 ± 0.94
Total triglycerides, mmol/L	1.44 ± 0.87	1.70 ± 1.17^*a*^	1.51 ± 0.88	1.60 ± 1.03^*a*^
Blood HbA1c, %	5.8 ± 0.6	6.0 ± 0.8^*a*^	6.0 ± 0.7^*a*^	6.0 ± 0.8^*a*^
Fasting plasma glucose, mmol/L	5.92 ± 0.98	6.27 ± 1.57^*a*^	6.17 ± 1.31^*a*^	6.19 ± 1.42^*a*^
2-h plasma glucose, mmol/L	6.37 ± 2.34	7.04 ± 2.88^*a*^	7.05 ± 2.69^*a*^	6.96 ± 2.75^*a*^
Fasting plasma insulin, mU/L	9.2 ± 9.1	13.4 ± 24.9^*a*^	12.5 ± 19.8^*a*^	12.4 ± 21.7^*a*^
2-hour plasma insulin, mU/L	53.9 ± 56.7	66.6 ± 64.6^*a*^	65.0 ± 59.3^*a*^	64.2 ± 60.6^*a*^
C-reactive protein, mg/L	2.07 ± 4.19	2.75 ± 4.18^*a*^	3.16 ± 5.58^*a*^	2.90 ± 4.87^*a*^
Alanine (mmol/L)	0.416 ± 0.064	0.433 ± 0.066^*a*^	0.428 ± 0.064	0.429 ± 0.065^*a*^
Glutamine (mmol/L)	0.514 ± 0.075	0.502 ± 0.073^*a*^	0.499 ± 0.075	0.502 ± 0.074^*a*^
Glycine (mmol/L)	0.277 ± 0.071	0.279 ± 0.072	0.278 ± 0.076	0.279 ± 0.075
Histidine (mmol/L)	0.064 ± 0.009	0.063 ± 0.009	0.062 ± 0.009^*a*^	0.063 ± 0.009^*a*^
Isoleucine (mmol/L)	0.060 ± 0.017	0.064 ± 0.019^*a*^	0.062 ± 0.017	0.063 ± 0.018^*a*^
Leucine (mmol/L)	0.090 ± 0.017	0.093 ± 0.02^*a*^	0.091 ± 0.017	0.092 ± 0.019^*a*^
Phenylalanine (mmol/L)	0.075 ± 0.012	0.080 ± 0.012^*a*^	0.080 ± 0.014^*a*^	0.080 ± 0.013^*a*^
Tyrosine (mmol/L)	0.057 ± 0.012	0.060 ± 0.012^*a*^	0.059 ± 0.011	0.059 ± 0.012^*a*^
Valine (mmol/L)	0.217 ± 0.037	0.221 ± 0.04	0.220 ± 0.037	0.220 ± 0.038

Values shown as mean ± SD for each category.

Abbreviations: CAD, coronary artery disease; CVD, cardiovascular disease; HDL, high-density lipoprotein; LDL, low-density lipoprotein; METSIM, Metabolic Syndrome in Men.

^*a*^Statistically significant differences (*P* < 0.002) between CAD, stroke, and CVD and control group (no CVD).

**Figure 1.**
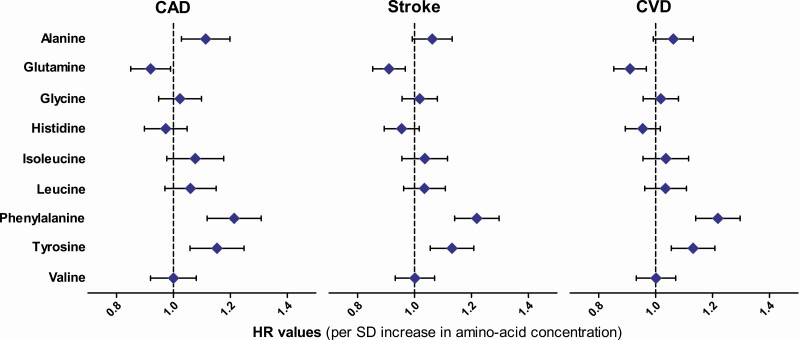
Hazards ratios (HR) and their 95% CIs for the associations of 9 amino acids with the risk of coronary artery disease (CAD), ischemic stroke, and cardiovascular disease (CVD) events (N = 9584). HR values were adjusted for age, diabetes status, fasting insulin, smoking, body mass index, low-density lipoprotein cholesterol, drug treatment for hypertension, high-sensitivity C-reactive protein, high-density lipoprotein cholesterol, and statin treatment.

Figure 1 and Supplementary Table 2 give the results for Cox regression analyses of the associations of amino acids with incident CAD, ischemic stroke, and CVD events (28). In unadjusted Cox regression analysis, alanine, isoleucine, leucine, phenylalanine, and tyrosine were significantly associated with increased risk of CAD, and glutamine and histidine with decreased risk of CAD. When adjusted for conventional cardiovascular risk factors, phenylalanine (HR 1.21; 95% CI, 1.12-1.31; *P* = 5.0-E06) and tyrosine (HR 1.15; 95% CI, 1.06-1.25; *P* = 0.002) remained significantly associated, and alanine nominally associated (HR 1.11; 95% CI, 1.03-1.20; *P* = 0.021) with an increase in CAD events, and glutamine nominally associated (0.92; 95% CI, 0.85-0.99; *P* = 0.026) with a decreased risk of CAD events.

In nonadjusted Cox regression analyses, alanine, phenylalanine, and tyrosine were significantly associated with increased risk of ischemic stroke, and glutamine and histidine significantly associated with decreased risk of ischemic stroke events (Fig. 1, 28). After the adjustment for confounding factors phenylalanine (HR 1.24; 95% CI, 1.13-1.37; *P* = 1.5E-05) was significantly associated with increased risk of ischemic stroke, and glutamine (HR 0.86; 95% CI, 0.78-0.95; *P* = 0.003) significantly associated with decreased risk of stroke (28). We also calculated the phenylalanine to tyrosine ratio reflecting phenylalanine hydroxylase activity. This ratio has been suggested to be a risk factor for acute ischemic stroke (29). Phenylalanine to tyrosine ratio was nominally associated with incident ischemic stroke in unadjusted model (HR 1.14; 95% CI, 1.04-1.25; *P* = 0.008) but lost its statistical significance when adjusted for confounding factors.

In unadjusted Cox regression analysis, alanine, isoleucine, leucine, phenylalanine, and tyrosine were significantly associated with increased risk of CVD, and glutamine and histidine with decreased risk of CVD. When adjusted for confounding factors (Fig. 1, 28) phenylalanine (HR 1.22; 95% CI, 1.14-1.30; *P* = 4.5E-09) and tyrosine (HR 1.13; 95% CI, 1.06-1.21; *P* = 0.001) remained significantly associated with an increase in CVD events, and glutamine significantly associated with a decrease in CVD events (HR 0.91; 95% CI, 0.85-0.97; *P* = 0.004) (28).

We calculated the C-index first for model 1 including conventional risk factors for CVD events, and then we added amino acids phenylalanine, tyrosine, and alanine in model 2. C-index increased nonsignificantly from 0.694 to 0.697 for CAD (model 2). In corresponding models, C-index increased from 0.705 to 0.710 (*P* = 0.032) for stroke, and from 0.692 to 0.696 (*P* = 0.017) for CVD.

## Discussion

Our 12.3-year follow-up study including 9584 participants of the METSIM study showed that alanine, glutamine, histidine, isoleucine, leucine, phenylalanine, tyrosine, and valine were significantly or nominally associated with CAD, ischemic stroke, or CVD events.

Previous studies have shown that conventional risk factors, such as elevated LDL-C concentration, hypertension, smoking, diabetes, obesity, central obesity, and inflammation are associated with an increase in CVD events (30). However, these established risk factors explain approximately only one-half of the risk of incident CAD events, and therefore new risk markers for CVD are needed.

Our study shows that a nonessential amino acid alanine nominally increased the risk of CAD by 11% after the adjustment for confounding factors. This is a novel finding not reported in previous studies. Alanine has been shown to be associated with elevated blood pressure (31), insulin resistance (9), nonalcoholic fatty liver disease (32), and type 2 diabetes (9), which may explain at least in part its role as a risk factor for cardiometabolic diseases. In our study, alanine correlated positively and significantly with several other cardiovascular risk factors, including BMI, waist, systolic blood pressure, fasting and 2-hour glucose and insulin, hs-CRP, and decreased insulin sensitivity.

Branched-chain amino acids isoleucine, leucine, and valine are essential amino acids in humans and play an important role in the pathogenesis of metabolic disorders, including obesity and diabetes (33). In our study, isoleucine and leucine were significantly associated with increased risk of CAD in an unadjusted model but after adjustment for confounding factors the associations lost their statistical significance. None of the branched-chain amino acids was significantly associated with ischemic stroke.

Several studies have been previously published about the role of branched-chain amino acids as risk factors for CVD. These studies have had differences in the design of the study (case-control vs prospective), the size of the study, and the definition of cardiovascular events (CAD vs combination of CAD and ischemic stroke). Almost all studies reported an association of branched-chain amino acids with CAD or CVD (11-17), but 1 previous study from Finland (18) failed to show that branched-chain amino acids are associated with increased risk of CVD events.

Phenylalanine is an essential aromatic amino acid, and the precursor for tyrosine and dopamine-related neurotransmitters. In our study, phenylalanine was the only amino acid significantly associated with CAD, ischemic stroke, and CVD events after the adjustment for confounding factors, confirming the results of a previous large prospective study comprising 3 population-based cohorts (18). Furthermore, phenylalanine has been shown to be associated with incident heart failure (34). In our study, phenylalanine correlated with elevated BMI, waist circumference, systolic blood pressure, LDL-C, total triglycerides, fasting and 2-hour glucose, insulin, and hs-CRP (35), and inversely with Matsuda ISI, all important cardiovascular risk factors. Additionally, we have previously shown that phenylalanine increased the risk of T2D by impairing both insulin secretion and insulin sensitivity (9). Interestingly, a recent study reported that a downstream gut microbial metabolite of phenylalanine, phenylacetylglutamine, was also associated with an increased risk of CVD especially in individuals with T2D (36).

Tyrosine is a nonessential amino acid and a precursor of the catecholamines. In our study, tyrosine was associated with increased risk of CAD by 15% and CVD by 13% after the adjustment for confounding factors. Tyrosine has been previously associated with an increase of carotid intima-media thickness in the Cardiovascular Risk in Young Finns Study (37). In our study, tyrosine was not associated with ischemic stroke. In our previous study, tyrosine was significantly associated also with an increase in the risk of T2D (9).

Histidine was significantly associated with a decreased risk of CAD, ischemic stroke, and CVD in an unadjusted Cox model in our study but after adjustment for confounding factors these associations were not statistically significant. Previous studies have reported that histidine is associated with a decreased risk of CAD (14, 15, 38) and ischemic stroke (38).

Glutamine is a nonessential amino acid and is among the most abundant amino acids in humans. Diet is the main source of nutrients involved in glutamine metabolism (39). In our study, glutamine decreased significantly ischemic stroke by 14% and CVD events by 9% after adjustment for confounding factors. Glutamine has a cardioprotective effect against CAD and CVD, as shown by a study including 2 cohorts, the Nurse’s Health Study and Health Professionals Follow-up study. This study showed that glutamine was inversely and significantly associated with cardiovascular mortality (40). As far as we know, the preventive effect of glutamine for ischemic stroke has not been previously published.

Our study demonstrates that metabolic risk factors (diabetes, fasting insulin, BMI, LDL-C, HDL-C) are important to take into account when analyzing the associations of amino acids with the risk of cardiovascular complications. Including metabolic risk factors in statistical analyses decreased significantly statistical significance between amino acids and CAD, stroke, and CVD suggesting that not only classic risk factors, but also metabolic factors play an important role in the risk of cardiovascular complications.

We calculated the C-index first for conventional risk factors for CVD events, and then added amino acids phenylalanine, tyrosine, and alanine in the models. C-index increased nonsignificantly for CAD events, but for stroke and CVD an increase in C-index was nominally significant. These results suggest that these amino acids increased the prediction for stroke and CAD significantly, but the improvement was relatively small.

The strengths of our study include the large and homogeneous METSIM study population. We had detailed phenotype at baseline, carefully defined diagnoses for CAD, ischemic stroke and CVD events, and a long follow-up period. Additionally, we adjusted our results for multiple confounding factors and used a very conservative threshold for statistical significance to increase credibility of our study. The limitation of our study is that it included only middle-aged and elderly Finnish men, and that our results do not imply causality between amino acids and cardiovascular events. We could not include all 20 amino acids in our statistical analyses because of limitations of the proton NMR method. Further studies in other populations are warranted to confirm our results and to assess their applicability to women and other populations and age groups.

In summary, our 12.3-year follow-up study of the METSIM cohort identified alanine as a new amino acid associated with increased risk of CAD and glutamine as a new amino acid associated with decreased risk of ischemic stroke. We also confirmed that phenylalanine and tyrosine were associated with CAD, ischemic stroke, and CVD events.

## Data Availability

Restrictions apply to the availability of data generated or analyzed during this study to preserve the confidentiality of the participants. The corresponding author will, on request, detail the restrictions and any conditions under which access to some data may be provided.

## Abbreviations

BMI: body mass index
CAD: coronary artery disease
CVD: cardiovascular disease
HR: hazard ratio
HDL-C: high-density lipoprotein cholesterol
hs-CRP: high-sensitivity C-reactive protein
ISI: insulin sensitivity index
LDL-C: low-density lipoprotein
METSIM: Metabolic Syndrome in Men
NMR: nuclear magnetic resonance
T2D: type 2 diabetes

## Additional Information

**Disclosure Statement:** The authors have nothing to disclose.
